# The hedgehog inhibitor GANT61 sensitizes prostate cancer cells to ionizing radiation both *in vitro* and *in vivo*

**DOI:** 10.18632/oncotarget.12483

**Published:** 2016-10-05

**Authors:** Annelies Gonnissen, Sofie Isebaert, Chad M McKee, Rüveyda Dok, Karin Haustermans, Ruth J Muschel

**Affiliations:** ^1^ KU Leuven, University of Leuven, Department of Oncology, Experimental Radiotherapy; University Hospitals Leuven, Radiation Oncology, Leuven, Belgium; ^2^ University of Oxford, Department of Oncology, CRUK/MRC Oxford Institute for Radiation Oncology, Oxford, UK

**Keywords:** prostate cancer, Hedgehog pathway, GANT61, radiotherapy, xenograft mouse model

## Abstract

Limited data exists regarding the combination of Hedgehog signaling (Hh) inhibition and radiotherapy, even though there are several indications that this might be a promising treatment strategy. In this study, we evaluated the combination of two Hh inhibitors, the SMO inhibitor GDC-0449 and the GLI inhibitor GANT61 with radiotherapy in different prostate cancer (PCa) models. *In vitro*, GANT61 was able to sensitize 22Rv1 PCa cells but not PC3 and DU145 PCa cells. The lack of radiosensitization in the latter cell lines was shown to be dependent on the presence of mutated p53. Introduction of WT p53 into PC3 cells resulted in radiosensization following GANT61 treatment, suggesting that the p53 transcription factor plays an important role in the GANT61-induced radiosensitization *in vitro*. Targeting at the level of SMO (GDC-0449) did not show cytotoxicity or synergy with radiation. Furthermore, we confirmed the radiosensitization effect of GANT61 in two *in vivo* xenograft PCa models. The decrease in tumor growth was associated with decreased proliferation and increased apoptosis. In conclusion, we provide evidence that GANT61 in combination with radiation treatment might represent a promising therapeutic strategy for enhancing the radiation response of PCa patients.

## INTRODUCTION

Prostate cancer (PCa) is the second most frequently diagnosed non-skin malignancy in men worldwide, with one in 16 afflicted men dying from this disease every year [1]. Despite the use of prognostic risk grouping systems and the multitude of treatment options that are available, including surgery, radiotherapy, hormonal therapy, biochemical relapse free survival rates as well as PCa-specific survival rates remain poor, especially in the higher risk categories [2, 3]. Hence, there is a need for novel treatment regimens. One option is the combination of radiotherapy with molecular agents that specifically target signaling molecules that play a key role in oncogenic processes while also modulating the response to ionizing radiation [4].

The Hedgehog (Hh) pathway is one of the body's major developmental pathways, and is of particular interest here as there is increasing evidence that this pathway is involved in PCa development, progression to more advanced disease states, and therapy-resistance [5, 6]. Canonical Hh signaling is activated when Hh ligands bind to the receptor Patched 1 (PTCH1) resulting in release of inhibition of the Hh regulatory protein Smoothened (SMO), which during Hh signaling moves to the primary cilium. The resulting activation of the Hh pathway allows Glioma-associated oncogene homolog (GLI) proteins to translocate to the nucleus and stimulate transcription of specific Hh pathway target genes.

There are limited published data regarding the combination of Hh signaling inhibitors and radiotherapy even though there are several indications pointing towards an interesting interplay between Hh signaling and radiation response that may be exploited therapeutically [5]. First, several preclinical studies have reported that Hh signaling is involved in radiation resistance of hepatocellular, pancreatic, esophageal and non-small cell lung cancer cell lines [7–10]. Second, a number of Hh pathway target genes are involved in processes that determine the radiation response (e.g. MYC, BCL2), and there is also crosstalk with other important oncogenic pathways that are known to influence the response to radiotherapy (e.g. PI3K, MAPK). Furthermore, there is evidence from clinical studies in patients with esophageal and cervical cancer that Hh activation after chemoradiotherapy is associated with poor outcome [10–13].

The importance of targeting Hh in cancer is currently gaining attention. A number of Hh inhibitors have been developed for clinical investigation, including those that primarily target the ligand SHH [14], and the pathway regulatory protein SMO. For example, the SMO inhibitor GDC-0449 has seen success in treating basal cell carcinoma [15]. Additionally, there have been reports that GDC-0449 is effective in changing vasculature in prostate cancer cells [16], suggesting that it could be of interest as an adjuvant therapy.

There is also interest in targeting signaling downstream of SHH and SMO, at the level of the GLI transcription factors. One reason is that inhibitors of SMO, located upstream of GLI1/2 in the pathway, have not proven to drive consistent clinical outcomes, either due to acquired resistance (as in medulloblastoma [17]) or to inherent resistance (e.g. in solid tumors, often characterized by non-mutation-driven Hh pathway activation) [18]. The best studied GLI inhibitor is GANT61, a small molecule that blocks transcription of essential Hh proteins [19]. However, limited data regarding its pharmacokinetic characteristics are available. Until now, only one recent study by Zhou *et al.* has investigated the combination of GANT61 and radiotherapy. In this study, a modest radiosensitizing effect of GANT61 was observed in renal cancer cells [20].

Here, we investigated the combination of Hh inhibition using GANT61 or GDC-0449 with radiotherapy in different PCa models both *in vitro* and *in vivo.* The aim was to demonstrate the potential of Hh inhibitors as an effective adjunct to radiotherapy and therefore investigate its promise as a therapeutic strategy for enhancing the radiation response of PCa patients.

## RESULTS

### Hedgehog signaling inhibition decreases prostate cancer cell viability more effectively by targeting GLI rather than SMO

**Figure 1:**
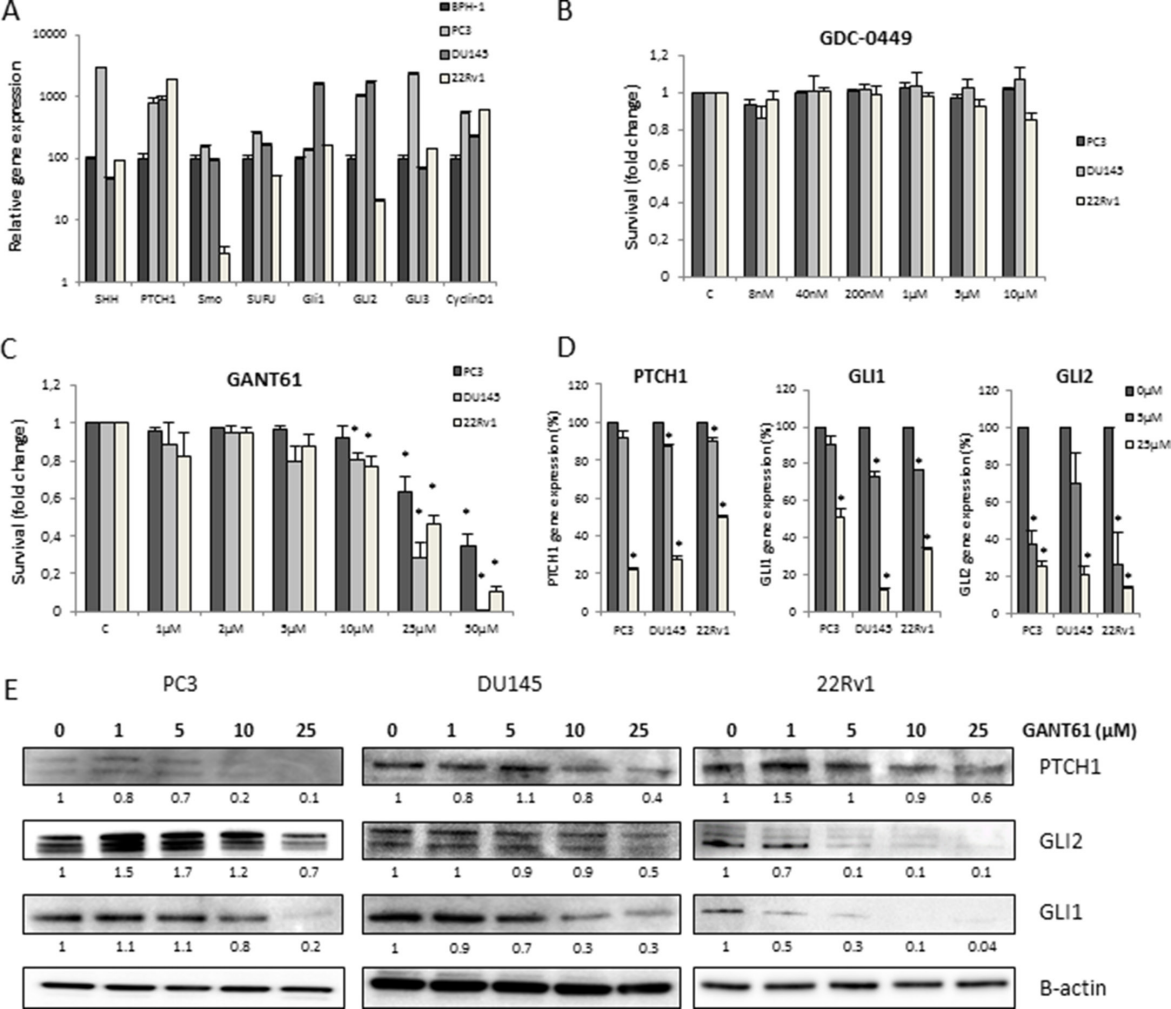
Hh inhibition in PCa cells (**A**) Gene profiling of Hh signaling in BPH-1 (black), PC3 (dark grey), DU145 (light grey) and 22Rv1 (white) PCa cell lines. Means ± SEM of 2 independent experiments performed in triplicate. (**B**, **C**) Cytotoxity after 72 h GDC-0449 (B) and GANT61 (C) in PCa cell lines. Means ± SEM of 3 independent experiments performed in quadruplicate. *p < 0.05 vs. control. (**D**) Changes in gene expression after 72 h treatment with GANT61 (5 μM/25 μM) of PTCH1, GLI1 and GLI2. Means ± SEM of 2 independent experiments performed in triplicate. *p < 0.05 vs. control. (**E**) Effect of 72 h GANT61 on protein expression of PTCH1, GLI1 and GLI2. Protein expression levels of indicated proteins were also assessed by means of densitometry (relative values indicated below the blots).

The gene expression of different Hh components was investigated in the benign prostate hyperplasia (BPH-1) cell line and three human PCa cell lines, i.e. the androgen-irresponsive PC3 and DU145 cells and the androgen-responsive 22Rv1 cells. Gene expression of GLI1 and PTCH1 were significantly higher in all PCa cell lines compared to the BPH-1 cells, illustrating the presence/relevance of Hh signaling in PCa (Figure 1A). Inhibition of Hh signaling (72 h) at the level of SMO using GDC-0449 (Vismodegib) did not have any significant effect on cell survival or proliferation in any of these PCa cell lines (Figure 1B and Figure S1A). However, inhibition downstream of SMO at the GLI1/2 proteins significantly decreased cell survival in a dose-dependent manner, when using the GLI-inhibitor GANT61 (Figure 1C). The reductions in proliferation observed in the presence of GANT61 persisted over several days (Figure S1B). GANT61 decreased both gene and protein expression of the Hh target genes PTCH1, GLI1 and GLI2, demonstrating the activity of the inhibitor (Figure 1D and Figure 1E). In contrast, we could not observe any effect of GDC-0449 on gene or protein expression of relevant Hh proteins (Figure S1C and Figure S1D).

### GANT61 increases radiosensitivity of 22Rv1 but not PC3 and DU145 prostate cancer cells

**Figure 2:**
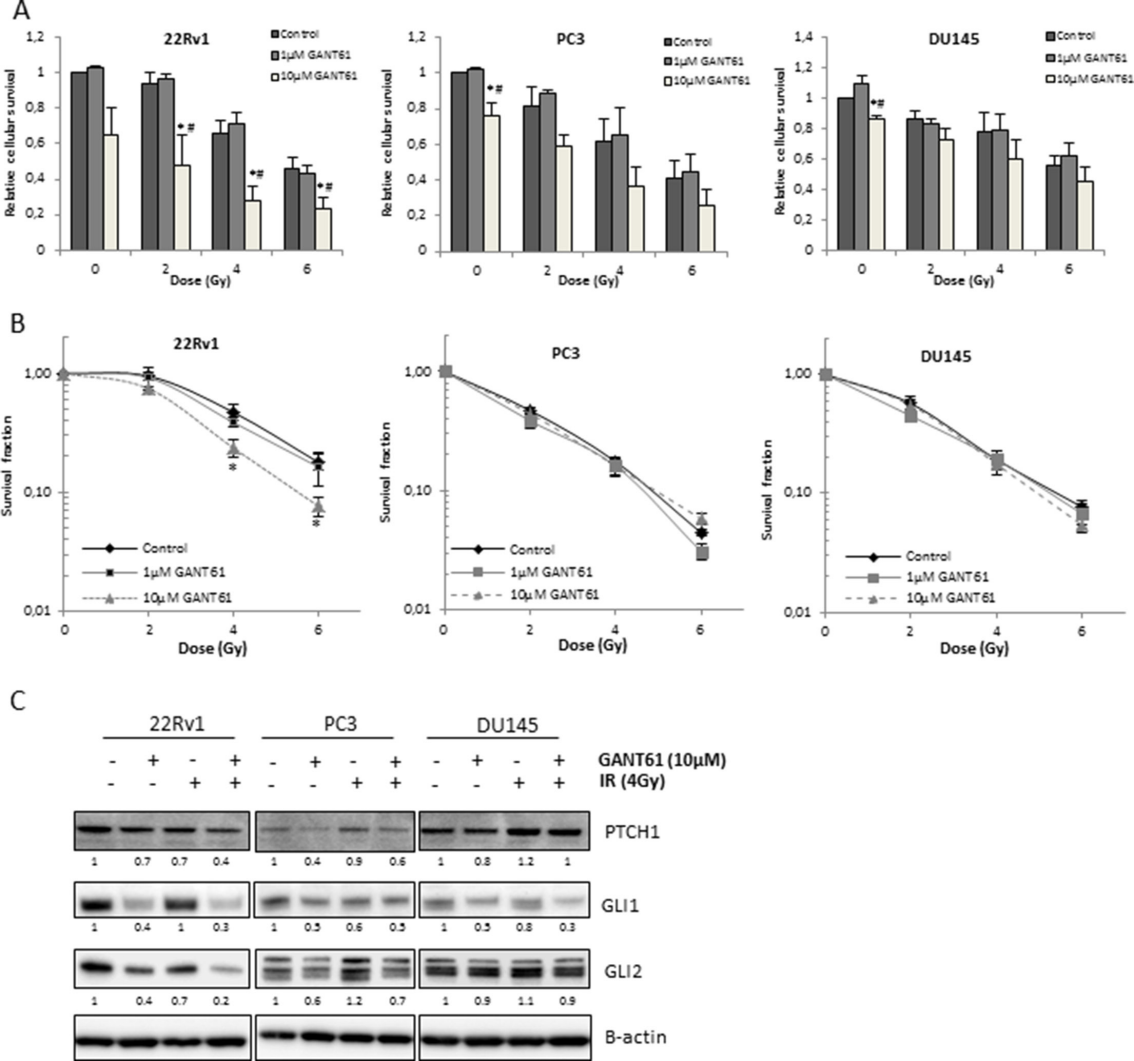
Effect of Hh inhibition on radiosensitivity of PCa cells (**A**) Relative cellular survival of the indicated cell lines determined by sulforhodamine B assay 7 days after treatment with increasing doses of ionizing radiation after 72 h pretreatment with GANT61. Means ± SEM of 3 independent experiments performed in quadruplicate. *p < 0.05 vs. control; ^#^*p* < 0.05 vs. GANT61. (**B**) Clonogenic survival curves after 72 h treatment with GANT61 (1μM/10μM) prior to/during IR. Means ± SEM of 3 independent experiments performed in triplicate. * < 0.05 vs. control. (**C**) Changes in PTCH1, GLI1 and GLI2 protein expression after GANT61 in combination with IR. Samples were pretreated with GANT61 (10 μM) for 72 h prior to IR (4 Gy) and proteins were isolated/lysed 24 h after IR. Protein expression levels of indicated proteins were also assessed by means of densitometry (relative values indicated below the blots).

To assess the effect of Hh inhibition in combination with ionizing radiation (IR) in PCa cells, short-term survival assays (Sulforhodamine B assays) were performed. GANT61 (10 μM) in combination with IR resulted in a decreased cell survival in all cell lines although only significant for 22Rv1 cells (Figure 2A). Next, clonogenic survival assays were performed to evaluate the effect of Hh inhibition on the intrinsic radiosensitivity of PCa cells (Figure 2B). The results showed that GANT61 (10 μM) significantly increased radiosensitivity of 22Rv1 cells (*p* = 0.002) with a dose-enhancement factor (DEF(0.5)) of 1.37 ± 0.09. In contrast, no significant effect of GANT61 on the radiosensitivity of PC3 or DU145 cells was observed (Figure 2B), even when a higher dose of 25 μM GANT61 was used (data not shown). Nevertheless, a significant reduction in PTCH1 and GLI1 gene and protein expression levels was observed in all cell lines after the combination treatment (Figure S2 and Figure 2C). GDC-0449 did not affect the radiosensitivity of any PCa cell line (Figure S3A and Figure S3B) in the same assays.

**Figure 3:**
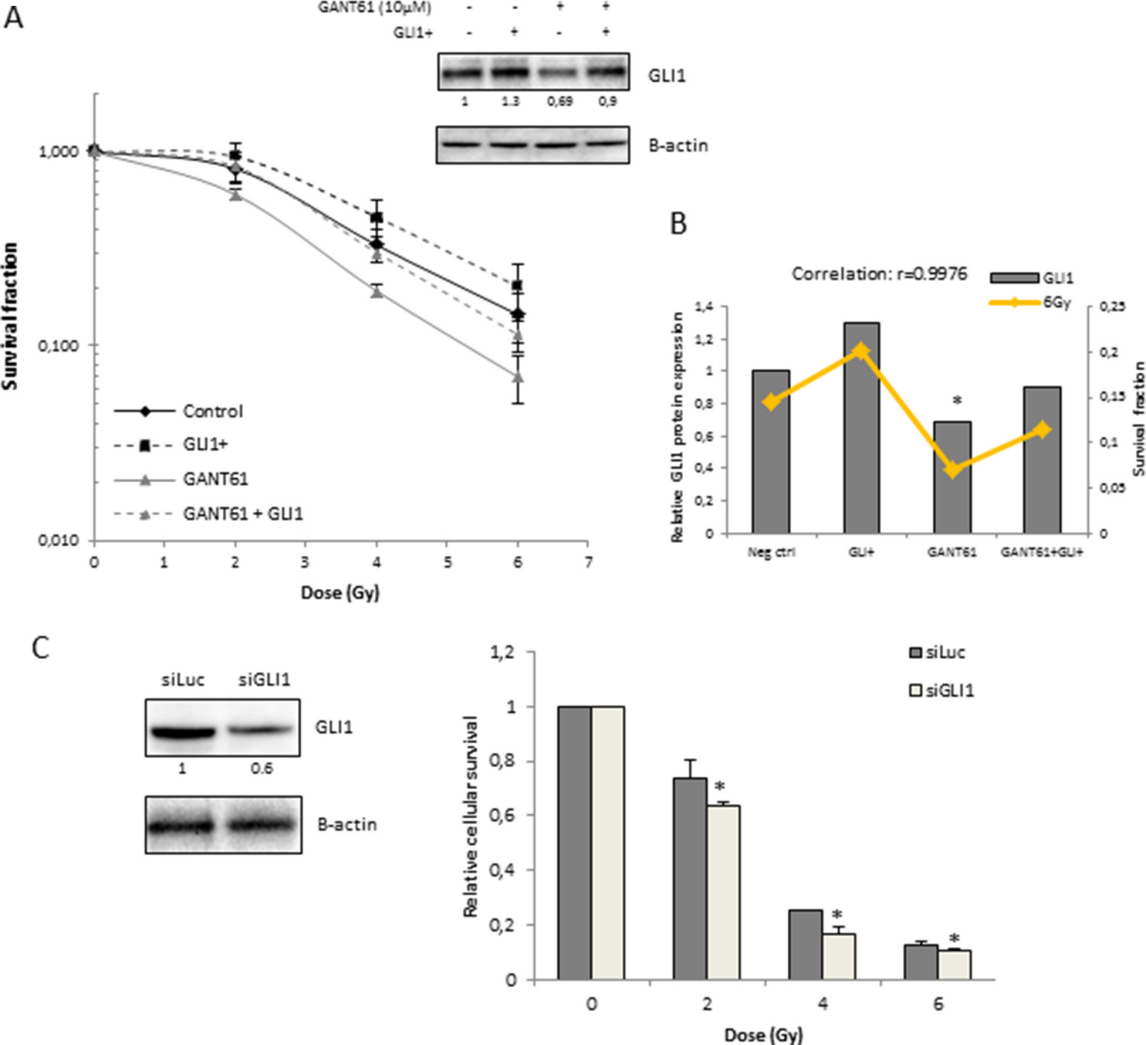
Role of GLI1 in radiosensitizing effect of GANT61 in 22Rv1 cells (**A**) Clonogenic survival curves after 72 h treatment with GANT61 (10 μM) prior to/during IR. 22Rv1 cells were transfected with GLI1 DNA 72 h prior to IR. Survival fraction was measured at 21 days after IR. GLI1 protein overexpression was validated with western blotting. Means ± SEM of 3 independent experiments performed in triplicate. (**B**) Correlation between the GLI1 protein levels (gray bars) and survival fraction of 22Rv1 cells after radiotherapy (6 Gy) in the different treatment groups. (**C**) Relative cellular survival of 22Rv1 cells determined by sulforhodamine B assay 7 days after treatment with increasing doses of ionizing radiation. Cells were transfected with siGLI1 for 72 h prior to IR. Knockdown of GLI1 was verified with western blotting and quantified by means of densitometry (relative values indicated below the blots). Means ± SEM of 2 independent experiments performed in triplicate. * < 0.05 vs. siLuc.

Next, we aimed to elucidate whether the radiosensitizing effect of GANT61 in the 22Rv1 cells was mediated by its effect on GLI1 since this is the main activator of Hh signaling. By overexpressing GLI1, we were able to counteract the GANT61-induced decrease in GLI1 protein expression. As a result, the radiosensitizing effect of GANT61 was repressed (Figure 3A). In addition, overexpression of GLI1 resulted in a radioprotective effect although this was not significant (*p* = 0.09). In line with this, the GLI1 protein level correlated with the survival fraction of 22Rv1 cells after IR (4 Gy: *r* = 0.9969 data not shown − 6 Gy: *r* = 0.9976 Figure 3B), indicating that the effect of GANT61 on the intrinsic radiosensitivity of these cells is (at least partially) due to targeted inhibition of GLI1. Furthermore, knockdown of GLI1 by siRNA silencing also decreased cell survival of 22Rv1 cells after IR (Figure 3C). These data indicate that GLI1 might play an important role in the response to radiation.

### GANT61 increases radiosensitivity of 22Rv1 cells primarily through inhibition of cell cycle and induction of apoptosis

**Figure 4:**
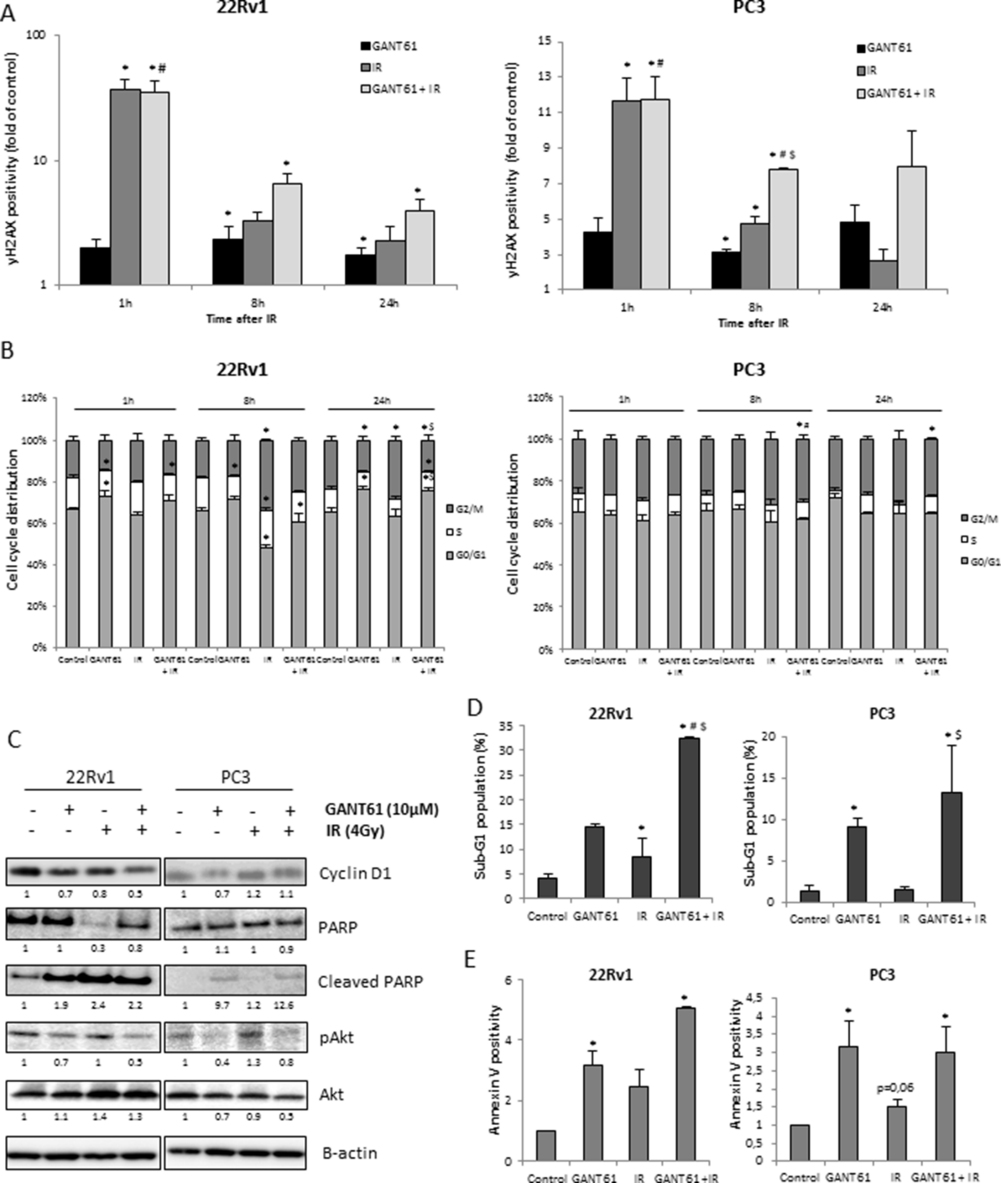
Radiosensitizing mechanisms of GANT61 (**A**) DNA damage response after 72 h treatment with GANT61 (10 μM) prior to/during IR (4 Gy). (**B**) Cell cycle distribution, (**C**) Cyclin D1, PARP, cleaved PARP, pAkt and Akt protein expression levels. Samples were pretreated with GANT61 (10 μM) for 72 h prior to IR (4 Gy) and proteins were lysed 24h after IR. Protein expression levels of indicated proteins were also assessed by means of densitometry (relative values indicated below the blots) (**D**) sub-G1 fraction (**E**) Annexin V-positive/PI-negative cells. For (A, B), cells were fixed at 1 h, 8 h and 24 h after IR and for (D, E), cells were analysed at 24 h after IR. Means ± SEM of 3 independent experiments. *p < 0.05 vs. control; ^#^*p* < 0.05 vs. GANT61; ^$^*p* < 0.05 vs. IR.

To investigate the effects of GANT61 on radiosensitivity, we evaluated its effects on DNA damage repair, cell cycle progression and apoptosis. Induction of DNA double strand breaks (DSB) immediately after IR, as indicated by γH2AX expression, was similar after IR or of IR in combination with GANT61. However, the combination treatment resulted in a significant delay in reduction in γH2AX expression indicating a reduction in DNA damage repair at 8 and 24 hours after IR in all three cell lines (Figure 4A and Figure S4A). Flow cytometric analysis indicated that GANT61 induced a G1 arrest in the 22Rv1 cells leading to a decreased amount of cells in the more radioresistant S-phase of cell cycle. Also noted were decreased protein levels of Cyclin D1 (Figure 4C), which can direct progression through the G1 phase of cell cycle. IR induced a G2/M arrest at 8h after IR, which was to some extent counteracted by GANT61, possibly due to the GANT61-induced G1 arrest (Figure 4B). Cell cycle alterations after IR were not perturbed by GANT61 in either PC3 or DU145 cells (Figure 4B and Figure S4B), although there was a slight decrease in Cyclin D1 expression (Figure 4C and Figure S4C).

Furthermore, the combination of GANT61 and IR in the 22Rv1 cells was associated with a significant increase of the sub-G1 fraction compared to either single treatment groups (Figure 4D), which was reflected in the results of cleavage of PARP (Figure 4C) and the Annexin- V^+^/PI^−^ measurements (Figure 4E) indicative of apoptosis. GANT61 also increased the sub-G1 fraction of PC3 and DU145 cells; however combination with IR did not further increase this population (Figure 4D, 4E and Figure S4D/S4E). In addition, GANT61 decreased expression of pAkt in all cell lines which could be associated with the decreased cell survival observed after 72 h GANT61 treatment (Figure 4C and Figure S4C).

These data indicate that inhibition of Hh signaling at the level of GLI1 increased the intrinsic radiosensitivity of 22Rv1 cells mainly through effects on cell cycle and apoptosis rather than DNA damage repair which was altered in all of the cell lines.

### GANT61-induced increase in radiosensitivity is mediated by p53 signaling *in vitro*

**Figure 5:**
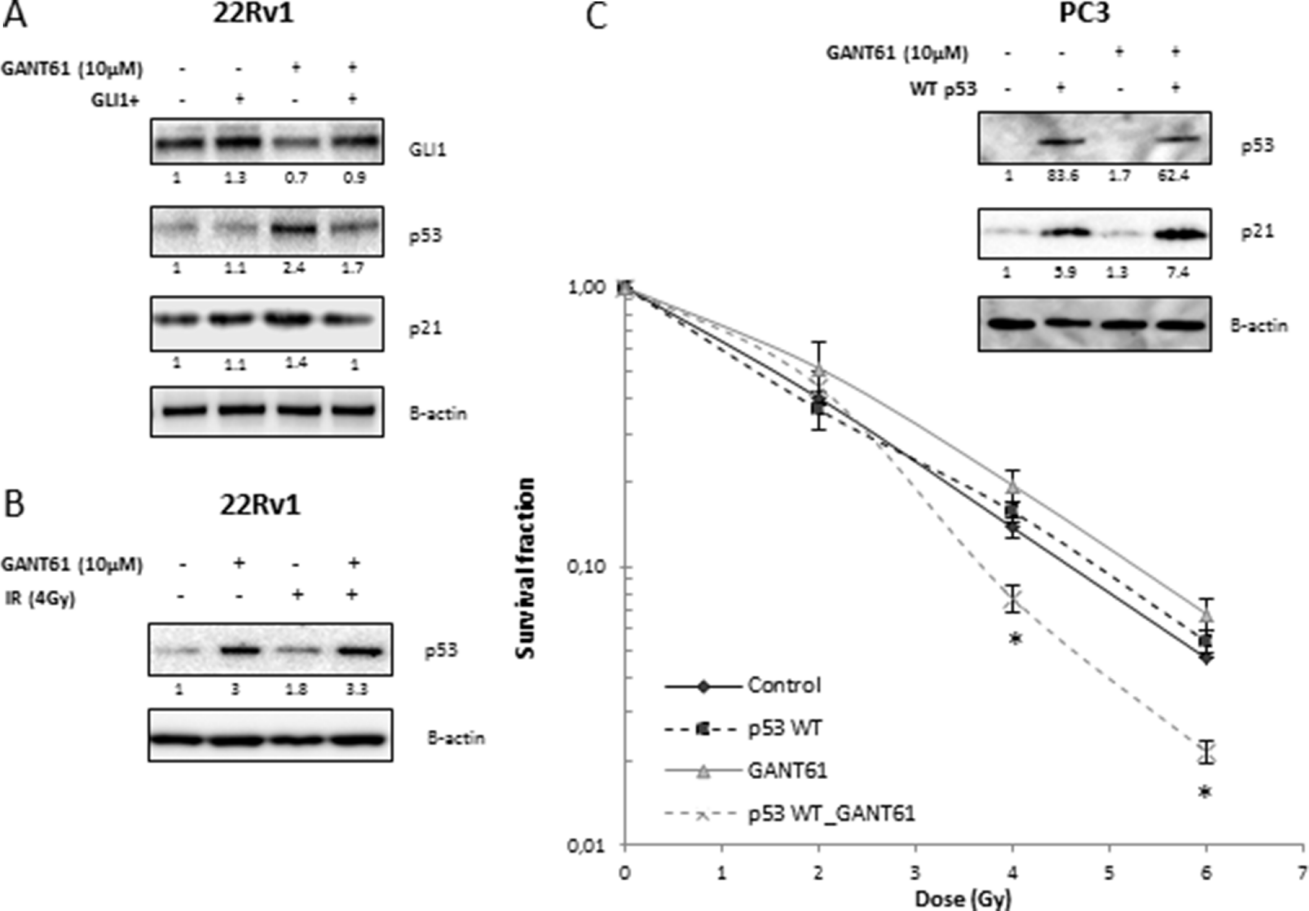
Role of p53 in radiosensitizing effect of GANT61 (**A**) p53 and p21 protein expression in 22Rv1 exposed to different treatment modalities. (**B**) p53 protein expression after GANT61 and/or IR. Samples were pretreated with GANT61 (10 μM) for 72 h prior to IR (4 Gy) and proteins were lysed 24 h after IR in 22Rv1 cells. (**C**) Clonogenic survival curves of PC3 cells after 72 h treatment with GANT61 (10 μM) prior to/during IR. PC3 cells were transfected with WT p53 48 h prior to IR. Means ± SEM of 3 independent experiments performed in triplicate. *p < 0.05 vs. control. p53 and p21 protein overexpression were validated with western blotting. Protein expression levels of indicated proteins were also assessed by means of densitometry (relative values indicated below the blots).

GANT61 enhanced the intrinsic radiosensitivity only of 22Rv1 cells and not that of PC3 or of DU145 cells. Both PC3 and DU145 cells harbor mutations in p53 whereas 22Rv1 cells have functional p53 [21]. GANT61 increased the protein expression levels of p53 and p21 in 22Rv1 cells. In contrast, overexpression of GLI1 in these cells decreased GANT61-induced expression of p53 (Figure 5A). IR alone also increased p53 expression (× 1.8), whereas the combination with GANT61 did not really enhance p53 expression compared to GANT61 alone (3.3 × compared to 3×) (Figure 5B). Based on these results, we hypothesized that p53 signaling might play an important role in radiosensitizion by GANT61. To further investigate this hypothesis, we introduced an expression vector for WT p53 into PC3 cells. Transfection of PC3 cells with WT p53 did not affect the radiosensitivity of PC3 cells, but resulted in radiosensitization after GANT61 treatment (Figure 5C). These data confirm that GANT61-induced radiosensitivity is likely dependent on p53 signaling in PCa cells.

### Concomitant GANT61 and radiotherapy synergistically reduced tumor growth *in vivo*

**Figure 6:**
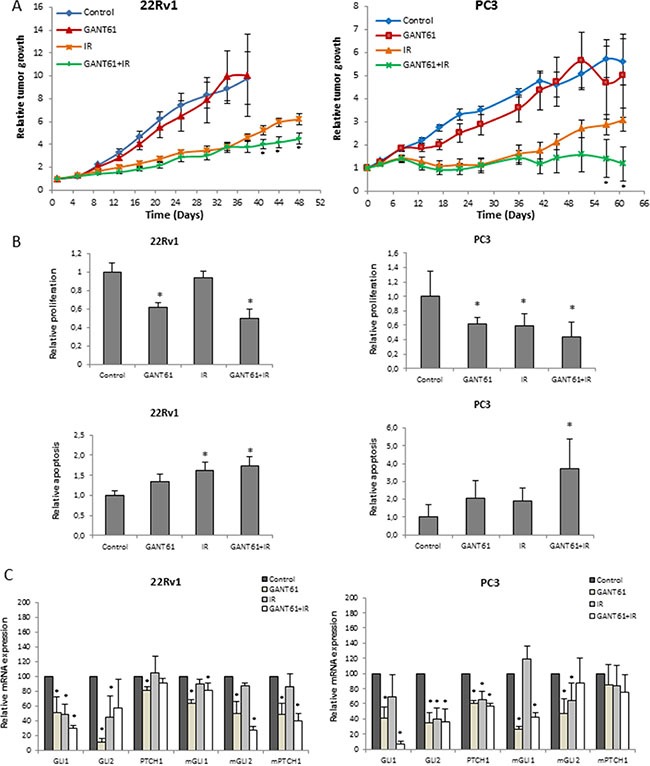
Effect of GANT61 and IR *in vivo* (**A**) Relative tumor growth of 22Rv1 (left) and PC3 (right) xenograft mice treated with concomitant GANT61 and IR (6 Gy) (6 mice per treatment group, ≤ 12 tumors). (**B**) Immunohistochemical analysis of Ki67 (upper) and cleaved caspase 3 (lower) immunostaining in the 22Rv1 (left) and PC3 (right) tumors. (**C**) qPCR analysis of Hh target genes (GLI1, GLI2 and PTCH1) in the 22Rv1 (left) and PC3 (right) tumors. Human and murine primers were used to assess gene expression in the tumor cells and surrounding stromal cells, respectively.

Next, the therapeutic potential of GANT61 in combination with IR was examined *in vivo.* First, a pilot study was performed to investigate the optimal treatment schedule when combining GANT61 and IR. Tumor growth was compared between the mice receiving the drug prior to IR, after IR or both before and after IR. Concomitant treatment of GANT61 and IR resulted in the greatest reduction of tumor growth compared to either single treatments or when GANT61 was only given before or after IR (Figure S5A). Interestingly, although we only observed a radiosensitizing effect of GANT61 in the 22Rv1 cells and not the PC3 cells *in vitro,* the combination of GANT61 and IR significantly reduced tumor growth in both xenograft tumor models in comparison with either single treatment (Figure 6A). Based on these promising results, we investigated the effect of GANT61 and IR in both 22Rv1 and PC3 xenograft models better understand better understand the underlying mechanisms of the radiosensitizing effect of GANT61.

Immunohistochemical analysis of the PCa xenograft tumors for Ki67 expression, which is a marker for proliferation potential, showed that GANT61 decreased Ki67 expression (*p* < 0.05) compared to controls (Figure 6B, upper panel). Ki67 expression in tumors treated with the combination of GANT61 and IR was less than after either single treatment; however, this trend was not significant. In line with this, expression levels of pERK and Cyclin D1 were also decreased in the combination group (Figure S5B). In addition, the combination treatment led to increased cleavage of caspase 3 compared to control (Figure 6B, lower panel). To evaluate the effect of GANT61 on tumor vasculature, we assessed mean vessel density (MVD) by counting the number of blood vessels (CD31^+^). Our data showed that MVD was decreased after GANT61 treatment, however not significantly in the PC3 xenograft model (Figure S5C). In the 22Rv1 xenograft model, we did observe a significant decrease in MVD in the tumors treated with the combination treatment. GANT61 or IR alone did not have an effect on MVD in this model (Figure S5C). We did not observe any significant changes in necrosis or hypoxia between the different tumor groups (Figure S5C). Thus, while changes in vasculature are potentially driven by GANT61 and IR treatment, reduced proliferation combined with elevated apoptosis appears to contribute most to the radiosentization effect.

To further characterize the effect of GANT61 in the tumors, we performed qPCR analysis using specific human and murine primers to distinguish the effect on the tumor cells and surrounding stromal cells, respectively. We were able to demonstrate that GANT61 alone or in combination with IR significantly reduced gene expression of the main Hh target genes GLI1, GLI2 and PTCH1 in the tumor cells, but also in the surrounding stroma (Figure 6C).

## DISCUSSION

In this study, we investigated the combination of Hh inhibition and radiotherapy in several PCa models both *in vitro* and *in vivo*. Our data demonstrate that targeting Hh signaling at the level of the GLI transcription factors is more effective than targeting it at the level of SMO in prostate cancer, as demonstrated using the GANT61 and GDC-0449 inhibitors respectively. Benvenuto *et al.* made similar observations in breast cancer models [22]. Moreover, several other studies have also shown a superiority of targeting Hh signaling more downstream in the signaling cascade [19, 23, 24]. One possible explanation is that the Hh signaling pathway can be activated by non-canonical oncogenic pathways downstream of SMO, which would make cell lines utilizing these pathways less sensitive to SMO inhibition. The presence of the primary cilium might also play a major role, as it is crucial for canonical Hh signaling activation. In the presence of Hh ligands, SMO will move to the primary cilium and activate the GLI transcription factors [25, 26]. However, the primary cilium is often lost in prostate cancer cells [27, 28], indicating pathway activation downstream of the cilia. This could explain the ineffectiveness of targeting the Hh signaling pathway at the level of SMO, i.e. upstream of the cilia. In addition, GANT61 has a broad spectrum of potential mechanisms (either through specific inhibition of Hh signaling or not) by which it could elicit anti-cancer effects as it targets many of the ‘classical hallmarks of cancer’ [29].

While several studies have already demonstrated that there might be a link between Hh signaling and radiation resistance, the specific mechanisms involved are not completely understood. Only one recent study by Zhou et al. has investigated the combination of GANT61 and radiotherapy. They observed a modest radiosensitizing effect of GANT61 in renal cancer cells, which was more pronounced after combination with HIF2 targeting. The authors showed that the effect of GANT61 on radiosensitivity was the result of decreased DNA damage repair in these cells [20]. Our data also showed that GANT61 delayed DSB repair after radiotherapy. However the GANT61-induced radiosensitizing effect seen in the prostate cancer cell lines and xenograft models could appear to be related to its effect on cell cycle distribution and apoptosis rather than DNA damage repair.

*In vitro*, these results pointed towards radiosensitization influenced by p53. It is well-known that p53 induces a p21-dependent G1-arrest and/or apoptosis in response to cellular stresses which are also important determinants of radiation response [30, 31]. A putative role of p53 was further supported by the fact that GANT61 only increased the radiosensitivity of the 22Rv1 cells and not PC3 and DU145 cells. This can be explained by the observation that only 22Rv1 cells possess functional p53, and our data showed that GANT61 activated p53 in the 22Rv1 cells. In addition, introduction of WT p53 into PC3 cells resulted in radiosensitization following GANT61 treatment. These data verify that the GANT61-induced radiosensitivity is elicited through p53 signaling *in vitro*.

Furthermore, we confirmed that increased radiosensitivity in 22Rv1 cells following GANT61 treatment is also partly mediated through specific inhibition of GLI1 as protein expression levels of GLI1 correlated with radiation sensitivity. Overexpression of GLI1 resulted in radioprotection of 22Rv1 cells although not significant (*p* = 0.09), whereas knockdown of GLI1 was associated with increased radiation sensitivity. This is in concordance with other preclinical reports that have shown that activated Hh signaling is associated with radiation resistance [7–10, 32].

A study by Stecca *et al.* has shown the presence of an inhibitory loop between GLI1 and p53 [33]. In this study, a negative correlation between GLI1 and p53 was observed which is in line with our results that show that GANT61 acts through inhibition of GLI1 and exerts its radiosensitizing effects via the downstream activation of p53 signaling and hence induction of cell cycle arrest and apoptosis.

Additionally, tumor growth rates in the combination group were significantly delayed compared to either single treatment groups in the xenograft models we interrogated. Our data *in vivo* demonstrate that the observed reduction in tumor size was associated with inhibition of proliferation and increased apoptosis. To our knowledge, this is first time that the combination of GANT61 and radiotherapy has been investigated in an *in vivo* setting.

While GANT61 increased the cell-intrinsic radiosensitivity of PCa cells *in vitro* in a p53-dependent manner through the induction of G1-arrest and apoptosis, the enhanced effect of radiotherapy due to GANT61 treatment *in vivo* seems to be independent of tumoral p53-status since radiosensitization was observed both in a PC3 and 22Rv1 xenograft model. An important factor in this respect might be the presence of a stromal compartment. Several reports have shown that PCa-associated stromal cells differ from normal prostate stromal cells. For example, tumor-associated stromal cells are able to induce tumorigenesis of benign prostate epithelial cells (BPH-1), whereas normal stroma did not [34, 35]. Wilkinson *et al.* found that active Hh signaling in these stromal cells was associated with increased proliferation and dedifferentiation of the adjacent epithelial cells, highlighting the importance of targeting Hh signaling in the tumor stroma [36]. Here, we indeed demonstrated that GANT61 inhibits Hh signaling both in the tumor cells and the surrounding stromal cells. This is also in line with the study by Zeng *et al.* that demonstrated that Hh inhibition at the level of SMO (HhAntag) enhanced radiation response of several lung cancer models by inhibition of paracrine stromal signaling rather than direct effect on the tumor cells itself [9]. In this context, it should be noted that Hh inhibition upstream the GLI transcription factors, such as SMO inhibition, could still be effective in an *in vivo* setting. A study by Karlou *et al.* has shown that GDC-0449 inhibits stromal Hh signaling in a MDA PCa 118b xenograft model [37].

In conclusion, we provide evidence that GANT61 in combination with radiation therapy could represent a promising therapeutic strategy for enhancing the radiation response of PCa patients. Further studies are required to understand the complex interaction between the stroma and tumor cells with regard to Hh inhibition to identify the patient populations that will most benefit from this combination strategy.

## MATERIALS AND METHODS

### Cell culture and drug exposure

The androgen-irresponsive prostate cancer cell lines PC3 and DU145 were purchased from the American Type Culture Collection (ATCC; Manassas, VA, USA). The PC3 cells were cultured in Minimal Essential Medium (MEM; Life Technologies, Carlsbad, CA, USA) supplemented with 10% fetal bovine serum (FBS; Life Technologies). The DU145 cells were cultured in MEM (Life Technologies) supplemented with 10% FBS, 1% non-essential amino acids (NEAA, Life Technologies) and 1% sodium pyruvate (Life Technologies). The androgen-responsive cell line 22Rv1 was purchased from the European Collection of Cell Cultures (ECACC) and cells were grown in RPMI 1640 medium without phenol red (Sigma Aldrich, St. Louis, MA, USA), supplemented with 10% FBS, 1% L-glutamine (Life Technologies) and 1% HEPES buffer (Life Technologies). The benign prostate hyperplasia (BPH-1) cells were a kind gift from prof. Swinnen (Lab of Lipid Metabolism and Cancer, KU Leuven) and were cultured in RPMI 1640 medium (Life technologies) supplemented with 10% FBS. All cells were maintained at 37°C in a humidified incubator with a 5% CO_2_/95% O_2_ atmosphere.

The GLI1/2 inhibitor GANT61 was purchased from Adipogen and Tocris (Bristol, UK) for the *in vitro* and *in vivo* experiments respectively. The SMO inhibitor GDC-0449 was obtained from Selleck Chemicals (Houston, TX, USA). Stock solutions were prepared in dimethyl sulfoxide (DMSO) and stored at −20°C. Working solutions were prepared immediately before use and control cells were treated with the corresponding drug solvent.

### Cell proliferation and survival

Cells were seeded in quadruplicate in a 96-well plate at a density of 2.5–45 × 10^3^ cells per well and were treated for 72 h with different concentrations of the inhibitors. Cell survival was assessed by means of a Sulforhodamine B (SRB) assay. Short-term survival assays were performed by pretreating the cells with GDC-0449 (1 μM/10 μM) or GANT61 (1 μM/10 μM) for 72 h followed by IR (2 Gy, 4 Gy, or 6 Gy). 24 h thereafter, fresh medium was added and cell survival was assessed 7 days later by means of SRB assay. Cell proliferation was measured using the Incucyte Zoom system (Essen BioScience, MI USA).

### Clonogenic cell survival assay

Cells were seeded in a 6-well plate at 7.5–30 × 10^4^ cells per well and the next day drug or solvent was added. After 72 h incubation with drug, cells were trypsinized and plated in triplicate at low density in 6-well plates. When cells were attached (after 4 h), they were irradiated with 2, 4 and 6 Gy using a Baltograph (Balteau NDT, Hermalle-sous-argenteau, Belgium) or mock irradiated. Medium was changed 16 hours post-irradiation. After 11–21 days, cells were fixed with 2.5% glutaraldehyde in PBS and stained with 0.4% crystal violet. The colonies containing 50 cells or more were counted with ColCount colony counter (Oxford Optronix, Oxford, UK). Survival fractions were calculated after normalizing for drug-induced toxicity. Dose-enhancement factor (DEF0.5) was calculated as the ratio of the dose needed for the control cells to the dose needed for the treated cells to reach a survival fraction of 0.5.

### Quantitative Real-time PCR (qPCR)

RNA isolation was performed using the Invitrap Spin Cell RNA Mini Kit from Stratec (Berlin, Germany) according to manufacturer's protocol. The RNA concentration was measured with a NanoDrop™ Lite Spectrophotometer from Thermo Scientific (Waltham, USA) RNA was subsequently reverse transcribed to cDNA with the SuperScript VILO cDNA synthesis kit (Life Technologies) and qPCR reactions were performed on the Lightcycler 480 (Roche) using Lightcycler 480 Sybr Green I Master mix (Roche).

Forward and reverse primer sequences for GAPDH, SHH, PTCH1, SMO, SUFU, GLI1, GLI2, GLI3 and CCND1 are enlisted in Table 1. Gene expression was calculated as expression per 100.000 copies of the household gene GAPDH.

**Table 1:** Forward and reverse primer sequences

Gene	Forward 5′ - 3′	Reverse 5′ - 3′
**hGAPDH**	CCATCTTCCAGGAGCGAG	TGAAGACGCCAGTGGAC
**hSHH**	CCCGACATCATATTTAAGGATGAAGA	AAGCGTTCAACTTGTCCTTAC
**hPTCH1**	AAACAGGTTACATGGATCAGATAATAG	CCCTTCCCAGAAGCAGT
**hSMO**	ACCTATGCCTGGCACACTTC	GTGAGGACAAAGGGGAGTGA
**hGLI1**	AATGCTGCCATGGATGCTAGA	GAGTATCAGTAGGTGGGAAGTCCATAT
**hGLI2**	GCCCTCACCTCCATCAAT	TGTTCTGGTTGGTGTCACT
**hGLI3**	GTGCTCCACTCGAACAGA	TCCAGGACTTTCATCCTCATTAGA
**hSUFU**	CCATGAGTTTACAGGAACAGAT	GTGCCAAGCCCTGCATTA
**hCYCLIN D1**	TGTAGTCACTTTATAAGTCATTG	CTTCAGCCATGAATAAGG
**mGAPDH**	TCAAGCTCATTTCCTGGTATGAC	TTACTCCTTGGAGGCCATGT
**mPTCH1**	CAAAGCCAAGGTTGTGGTAAT	TCTCACTCGGGTGGTCC
**mGLI1**	GCACGTTTGAAGGCTGTC	CTTCTCACCCGTGTGCGA
**mGLI2**	CACTCCAATGAGAAACCCTAC	CAGTCTTCACATGCTTGCG

### Immunoblot analysis

Protein lysates were made using RIPA lysis buffer containing phosphatase inhibitors (phenylarsine oxide and sodium orthovanadate; Sigma) and protease inhibitor cocktail (Roche). Protein concentrations were determined with the Bradford assay (Bio-Rad Laboratories, Hercules, CA, USA). Equal amounts of protein were separated on NuPage gels (Life Technologies) and blotted on PVDF membrane (Bio-Rad Laboratories). Membranes were blocked with 5% non-fatty dry milk (NFDM) in TBS-T for 1 hour followed by incubation with the primary antibody overnight at 4°C. Immunoblotting was performed with primary antibodies against ERK (#4695, 1:1000), pERK (#9106, 1:500) GLI1 (#2534, 1:500), PARP (#9532, 1:1000), cleaved PARP (#9541, 1:1000), SHH (#2207, 1:1000), SUFU (#2520, 1:1000) from Cell Signaling Technologies (Beverly, MA, USA), PTCH1 (sc-6149, 1:200) from Santa Cruz (Dallas, TX, USA), GLI2 (600-401-845, 1:1000) from Rockland Immunochemicals (Limerick, PA, USA) and SMO (SAB1404382, 1:500) from Sigma. Β-actin (Cell Signaling Technologies, #4967, 1:1000) was used as loading control. After incubation with the appropriate HRP-conjugated secondary antibody against rabbit (Cell signaling Technologies, #7074,1:3000), mouse (GE Healthcare, NA931, 1:300) or goat (Santa Cruz, sc2020, 1:3000), membranes were incubated with an enhanced chemiluminescence detection system (Perkin Elmer, Waltham, MA) and immune-reactive proteins were visualized using Fujifilm LAS-3000 mini camera (Fujifilm, Düsseldorf, Germany). Densitometry analysis was performed using ImageJ Software.

### Animal experiments

Animal experiments were approved by the local ethics committee of either the University of Oxford or KU Leuven (P131/2014). Male NMRI Nu/Nu mice (Janvier, Saint Berthevin, France) were inoculated with 2.5 × 10^5^ PC3 cells in 50 μl medium with 50 μl Matrigel (VWR, Radnor, PA, USA). Once these mice had palpable tumors (~150 mm³), they were divided into different experimental groups (*n* = 6/group). First a pilot experiment was performed to evaluate the optimal treatment schedule. GANT61 (30 mg/kg) was given every other day via oral gavage for two weeks. At day 7, a single dose of IR (6 Gy) was administered to the tumor. Tumor growth was compared between the mice receiving the drug either only before IR, either only after IR or concomitant with IR (before and after IR) (*n* = 6). The most effective treatment schedule, i.e. concomitant treatment, was then applied to the following experiment. GANT61 (oral gavage; 50 mg/kg body weight in 9:1 corn oil/ethanol solution; every other day for 2 weeks) was delivered both before and after IR (6 Gy at day 7) as indicated. For the 22Rv1 xenograft experiment, 2 × 10^6^ 22Rv1 cells were injected subcutaneously in both flanks of male NMRI Nu/Nu mice. When tumor volume reached 150 mm^3^, mice were treated with GANT61 (intraperitoneally; 50mg/kg body weight in 9:1 saline/ethanol solution). Tumors were irradiated at day 5 with a dose of 6 Gy. Tumor growth was followed by 3-weekly caliper measurements and tumor volumes were calculated (V = (l*b*h)*π/6). During drug treatment, the mice's body weight was measured as well. Mice were euthanized either at the end of GANT61 treatment or when the tumors reached the maximum ethically permitted volume of 2 × 10³ mm³. Pimonidazole was injected intraperitoneally 30 min before sacrificing the mice. Afterwards, all tumors were isolated and half of the tumor was fixed in formalin and fixed in paraffin for immunohistochemical analysis and the other half was snap frozen for protein analysis.

### Immunohistochemical analysis

After antigen retrieval and blocking (Supplemental Table S1), tumor sections were incubated overnight at 4°C with anti-pimonidazole (1/400, Hypoxyprobe, Massachusetts, USA), anti-caspase-3 (ready to use, Biocare Medical, Concord CA, USA) or anti-CD31 (1/25, Dianova, Hamburg, Germany), anti-GLI1 (1/50, Santa-Cruz), anti-GLI2 (1/1000, Rockland), anti-PTCH1 (1/300, Santa Cruz); or for 30 min at room temperature with anti-Ki67 (ready to use, Thermo Scientific). Appropriate secondary antibodies followed by 3.3′-diaminobenzidine (DAB) substrate (DAKO, Glostrup, Denmark) were used to visualize antigen presence.

Quantification of Ki67 was performed by counting the number of Ki67 positive nuclei in the tumor tissue. Mean vessel density (MVD) was assessed as the number of blood vessels (CD31^+^) per field for 10 high-power fields per tissue specimen. Tumor hypoxic fraction was determined as the percentage of cytoplasmic pimonidazole positive cells. The apoptotic fraction was determined by assessing the number of caspase-3 positive cells per tissue section.

### Overexpression GLI1 and p53

GLI1 plasmid (pLUT7 HA-GLI1) was a gift from Michael Ruppert (Addgene plasmid # 62970) [38]. p53 plasmid (pLU NP-wtp53) was a generous gift from Prof. Anna Sablina. Transient overexpression was performed using the Lipofectamin 2000^TM^ Reagent according to manufacturer's instructions. Overexpression of targeted proteins was controlled by means of western blotting.

### GLI1 silencing

The day before transfection, 22Rv1 cells were seeded at 5 × 10^5^ cells/ well in a 6 well plate. Transient silencing of GLI1 was performed using the Lipofectamin 2000^TM^ Reagent according to manufacturer's instructions. Following siRNAs against GLI1 were used: Ambion Silencer^®^ Select s5816, s5815 (Life Technologies) and ON-TARGETplus SMART pool, L-003896 (Dharmacon). Silencer^®^ Select Negative Control (4390843, Life Technologies) was used as a negative control. Knockdown of GLI1 was controlled by means of western blotting.

### Flow cytometry

#### Apoptosis (Annexin-V/PI)

Apoptotic cells were detected by means of Annexin-V-FLUOS detection kit (Roche Applied Science, Hague Road, IN, USA). Cells positive for Annexin-V and negative for propidium iodide were considered as cells in early apoptosis. The apoptotic fraction was measured with the FACSVerse Flow cytometer (BD Biosciences).

### DNA damage and cell cycle distribution

Cells were fixed with 70% ethanol and incubated for 2 h with Alexa Fluor^®^ 488 Mouse anti-H2AX (BD Biosciences, 560445, 1:20) in 0.25% Triton-X100. Afterwards, the cells were stained with 10 μg/ml propidium iodide containing 100 μg/ml RNase A. Cell cycle distribution and DNA damage was assessed with the FACSVerse Flow cytometer (BD Biosciences).

### Statistical analysis

For the *in vitro* experiments, a one-way ANOVA with Tukey's multiple comparison test or a two-tailed student's *t-test* was performed. For the analysis of *in vivo* experiments, the Kolmogorov-Smirnov method was used to test for normality. A two-tailed student's *t-test* was used when the data complied with the conditions of normality and equal variance. Under other conditions, comparisons were carried out by nonparametric analysis using the Mann–Whitney rank-sum test. Statistics were calculated using the software package Statistica 12 (StatSoft Inc, Tulsa, OK, USA). A *p-value* of < 0.05 was considered statistically significant.

## SUPPLEMENTARY MATERIALS FIGURES AND TABLES


