# Supplementary material for: The hedgehog inhibitor GANT61 sensitizes prostate cancer cells to ionizing radiation both in vitro and in vivo
Source: Oncotarget. 2016 Oct 5;7(51):84286–98. doi: 10.18632/oncotarget.12483 (PMC5356662; doi:10.18632/oncotarget.12483)
Supplement: Supplementary file 1 [file oncotarget-07-84286-s001.pdf]

The hedgehog inhibitor GANT61 sensitizes prostate cancer cells to ionizing radiation both *in vitro* and *in vivo*

Supplementary Materials

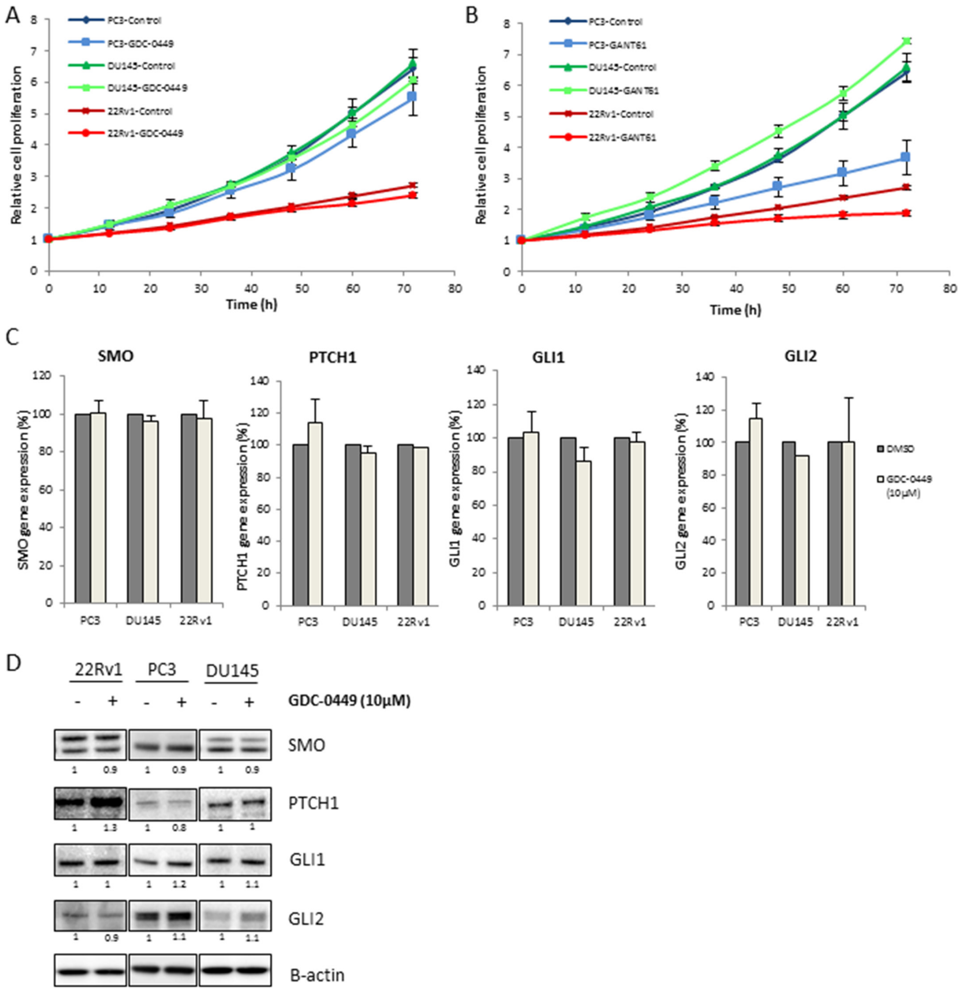

Supplementary Figure S1: Effect of Hh inhibition on PCa cell proliferation. (**A**, **B**) Cell proliferation after (A) GDC-0449 (10 µM) and (B) GANT61 (10 µM) in PCa cell lines using the Incucyte Zoom system. Means ± SEM of 3 independent experiments performed in quadruplicate. (**C**) Changes in gene expression after 72 h treatment with GDC-0449 (10 µM) of SMO, PTCH1, GLI1 and GLI2. Means ± SEM of 2 independent experiments performed in triplicate. (**D**) Effect of 72 h GDC-0449 on protein expression of SMO, PTCH1, GLI1 and GLI2. Protein expression levels of indicated proteins were also assessed by means of densitometry (relative values indicated below the blots).

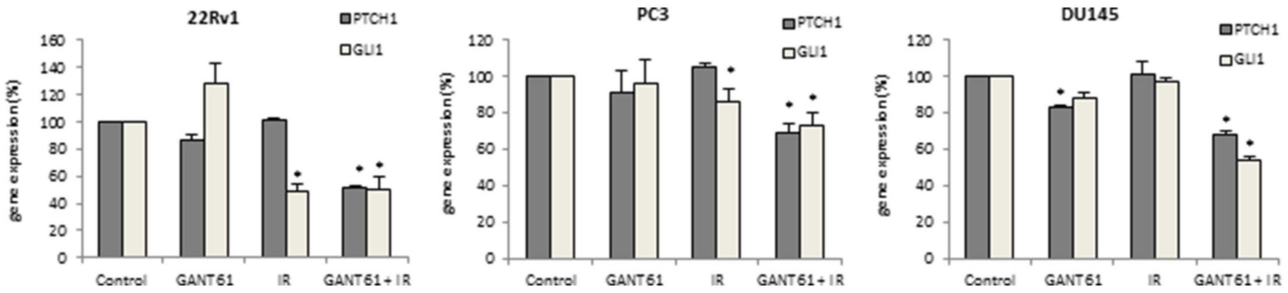

Supplementary Figure S2: Effect of GDC-0449 on Hh gene expression. (**A**) Changes in PTCH1 and GLI1 gene expression after GANT61 in combination with IR. Samples were pretreated with GANT61 (10 µM) for 72 h prior to IR (4 Gy) and RNA was isolated 24 h after IR. Means ± SEM of 2 independent experiments performed in triplicate. **p* < 0.05 vs. control.

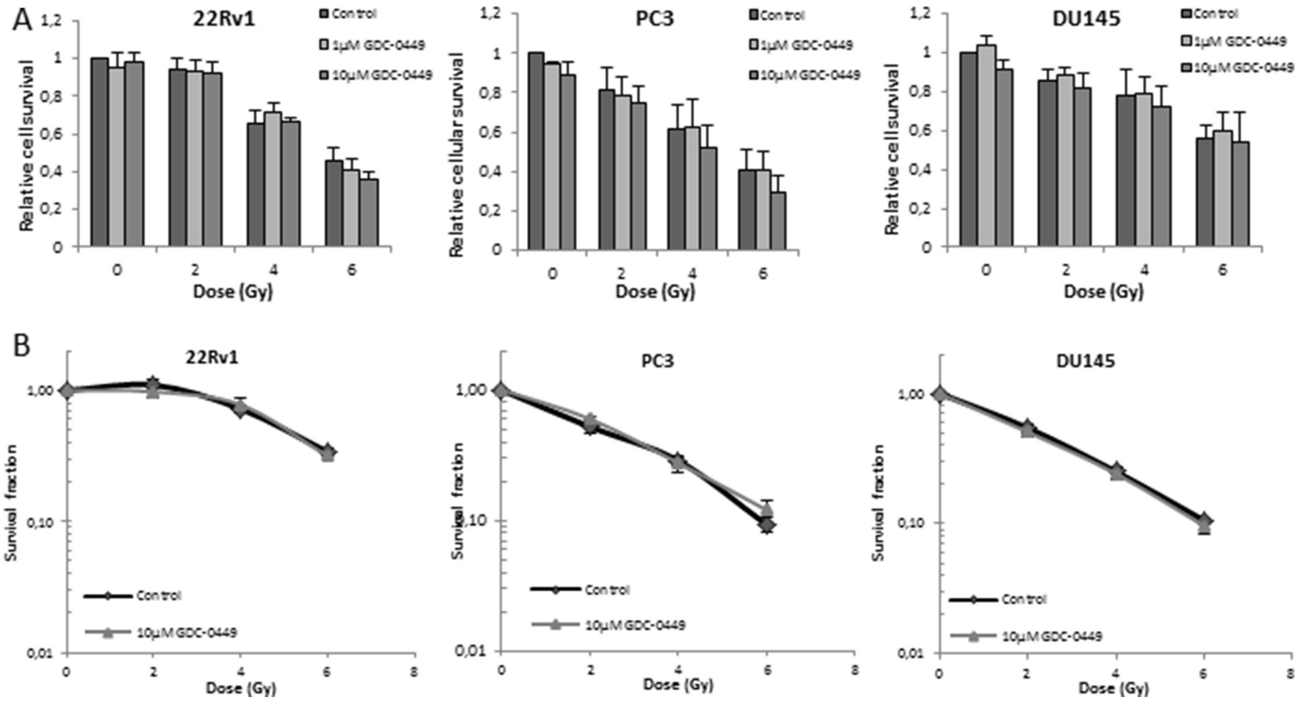

Supplementary Figure S3: Effect of GDC-0449 on radiosensitivity of PCa cells. (**A**) Relative cellular survival of the indicated cell lines determined by sulforhodamine B assay 7 days after treatment with increasing doses of ionizing radiation after 72 h pretreatment with GDC-0449 (1 µM/10 µM). Means ± SEM of 3 independent experiments performed in quadruplicate. (**B**) Clonogenic survival curves after 72 h treatment with GDC-0449 (10 µM) prior to/during IR. Means ± SEM of 3 independent experiments performed in triplicate.

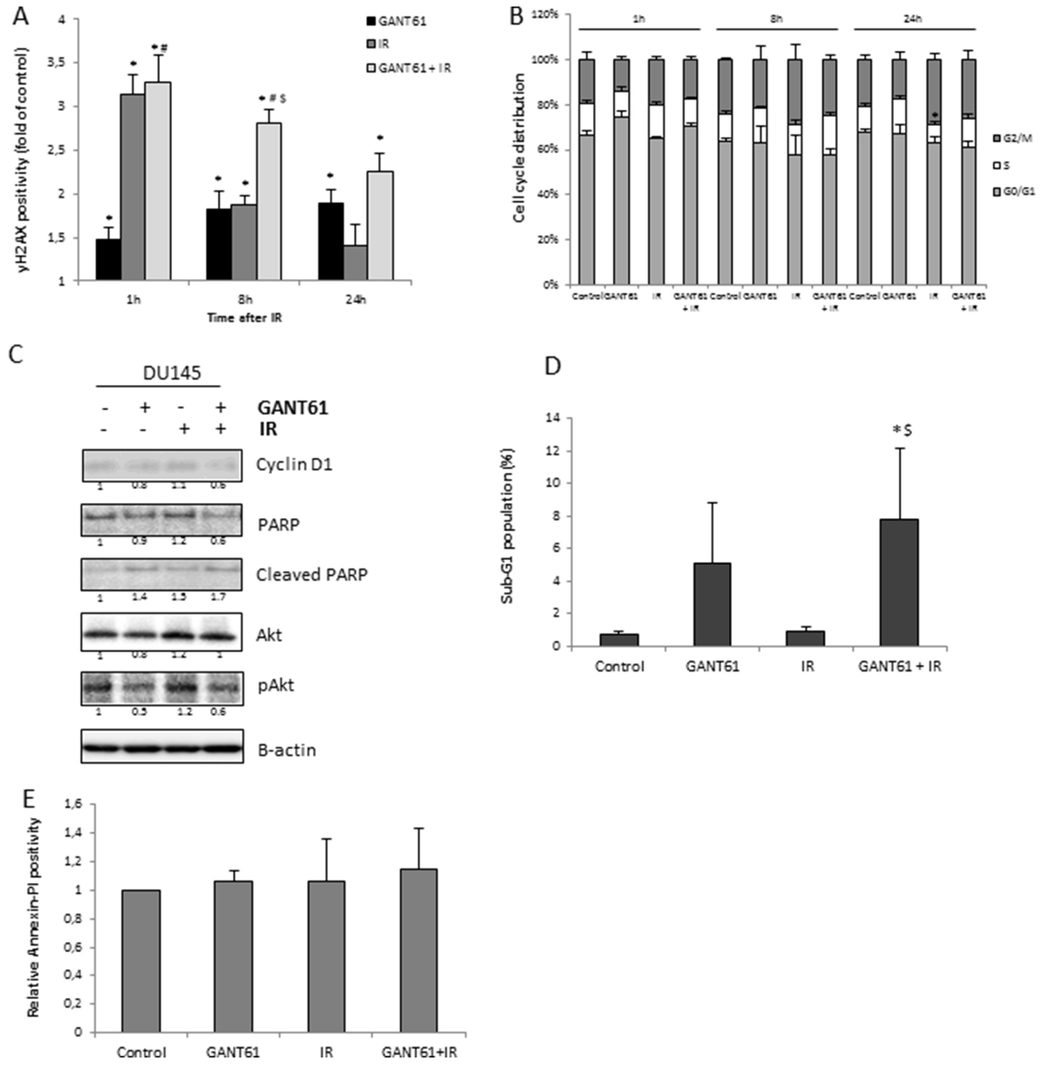

Supplementary Figure S4: Radiosensitizing mechanisms of GANT61 in DU145 cells. (**A**) DNA damage response after 72 h treatment with GANT61 (10 μM) prior to/during IR (4 Gy), (**B**) Cell cycle distribution, (**C**) Cyclin D1, PARP, cleaved PARP, pAkt and Akt protein expression levels. Samples were pretreated with GANT61 (10 μM) for 72 h prior to IR (4 Gy) and proteins were lysed 24 h after IR. Protein expression levels of indicated proteins were also assessed by means of densitometry (relative values indicated below the blots). (**D**) sub-G1 fraction, and (**E**) Annexin V-positive/PI-negative cells. For (A, B), cells were fixed at 1h, 8h and 24h after IR and for (D, E), cells were analysed at 24 h after IR. Means ± SEM of 3 independent experiments. *$p < 0.05$ vs. control; $^{\#}p < 0.05$ vs. GANT61; $^{\$}p < 0.05$ vs. IR.

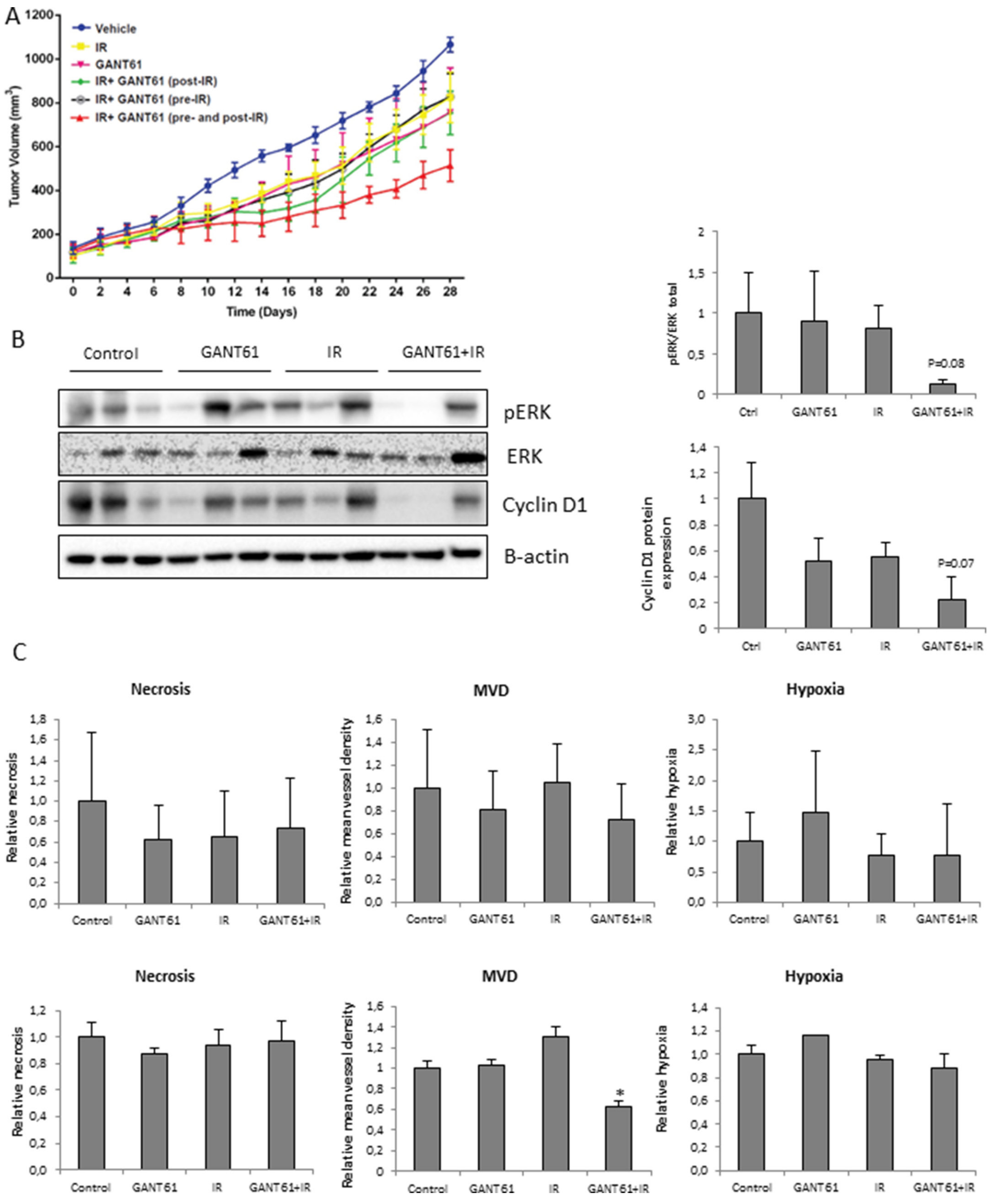

Supplementary Figure S5: Additional data of xenograft experiments. (**A**) pilot PC3 xenograft experiment to evaluate optimal treatment schedule. GANT61 (30 mg/kg) was given every other day via oral gavage for two weeks. At day 7, a single dose of IR (6 Gy) was administered to the tumor. Tumor growth was compared between the mice receiving the drug either only before IR, either only after IR or concomitant with IR (before and after IR) (n = 6). (**B**) Protein expression (left) of isolated PC3 tumors at the end of experiment (pERK, ERK, Cyclin D1) and densitometric quantification for the different bands (right). (**C**) Quantification of necrosis, microvessel density (CD31) and hypoxia (pimonidazole) of PC3 (upper panels) and 22Rv1 (lower panels) tumors isolated at the end of the experiment assessed by means of IHC (n = 4–8).

Supplementary Table S1: Specifications of antigen retrieval and antibodies used for immunohistochemical staining

	Head induced epitope retrieval	Primary antibody	Secondary antibody
**Ki67**	Tris-EDTA (Ph9)	Anti-human Ki67 rabbit (Thermo Scientific, RM-9106-R7), ready-to-use	Envision HRP Anti-rabbit (Dako), ready-to-use
**Pimonidazole**	Citrade buffer (0,01 M, pH6)	Anti-human piomonidazole rabbit (Hypoxyprobe, HP3-100kit), 1/100	Envision HRP Anti-rabbit (Dako), ready-to-use
**Cleaved caspase-3**	Reveal buffer Decloaker (1×) (Biocare Medical, Concord, CA, USA)	Anti-human Cleaved caspase 3 (RTU, Biocare Medical, PP 229 AA), ready-to-use	Envision HRP Anti-rabbit (Dako), ready-to-use
**CD31**	Citrate buffer (0,01 M, pH6)	Murine CD31 rat monoclonal (Dianova SZ31), 1/25	Biotinylated goat anti-rat lgG (Vector Labs), 1/100
**GLI1**	Citrate buffer (0,01 M, pH6)	Anti-human GLI1 rabbit polyclonaa (Santa Cruzsc-20687), 1/50	Envision HRP Anti-rabbit (Dako), ready-to-use
**GLI2**	Citrate buffer (0,01 M, pH6)	Anti-human GLI2 rabbit (Rockland 600-401-845 S), 1/1000	Anti-rabbit (Vector Labs), ready-to-use
**PTCH1**	Citrate buffer (0,01 M, pH6)	Anti-human PTCH1 goat (Santa Cruz sc-6147), 1/300	Anti-goat (Vector Labs), ready-to-use
